# Melatonin as a Pre- and Postharvest Tool for Enhancing Fruit Quality

**DOI:** 10.3390/plants15020331

**Published:** 2026-01-22

**Authors:** Pedro Antonio Padilla-González, Fernando Garrido-Auñón, María Emma García-Pastor, Fabián Guillén, María Serrano, Daniel Valero, Vicente Agulló

**Affiliations:** 1 Department of Food Technology, Escuela Politécnica Superior de Orihuela (EPSO) Instituto de Investigación e Innovación Agroalimentaria y Agroambiental (CIAGRO), University Miguel Hernández, Ctra. Beniel km. 3.2, 03312 Orihuela, Alicante, Spain; ppadilla@umh.es (P.A.P.-G.); fgarrido@umh.es (F.G.-A.); fabian.guillen@umh.es (F.G.); 2 Department of Applied Biology, Escuela Politécnica Superior de Orihuela (EPSO) Instituto de Investigación e Innovación Agroalimentaria y Agroambiental (CIAGRO), University Miguel Hernández, Ctra. Beniel km. 3.2, 03312 Orihuela, Alicante, Spain; m.garciap@umh.es (M.E.G.-P.); m.serrano@umh.es (M.S.)

**Keywords:** melatonin, preharvest, postharvest, plant hormones, fruit quality, cross-talk

## Abstract

Melatonin (MEL), also known as *N*-acetyl-5-methoxytryptamine, has been reported in plants as a secondary messenger involved in regulating abiotic stress responses. MEL treatment, either preharvest or postharvest, regulates several physiological and biochemical processes during fruit growth and ripening in horticultural products. These include reproductive development, tissue and quality maintenance, delayed senescence, and responses to abiotic stress. Due to its natural origin, low toxicity, and multifunctional regulatory capacity, MEL has recently attracted attention as a promising ‘green preservative’ for sustainable postharvest management. Additionally, MEL coordinates through cross-talk with other plant hormones, such as abscisic acid, ethylene, polyamines, jasmonic acid, γ-aminobutyric acid, salicylic acid, and nitric oxide, to regulate postharvest ripening and senescence. Furthermore, MEL enhances antioxidant systems and improves membrane integrity, thereby alleviating chilling injury and enhancing fruit firmness and colour. Notably, recent evidence highlights the innovative regulatory mechanisms of MEL involving redox homeostasis, hormone signalling reprogramming, and transcriptional modulation of stress-responsive pathways. MEL could therefore be considered an emerging, eco-friendly tool for prolonging the shelf-life of fruit and vegetables and maintaining their quality. This review summarises the mechanisms by which MEL contributes to plant stress resistance by regulating the biosynthesis and metabolism of stress tolerance and improving fruit quality.

## 1. Melatonin Biosynthesis and Its Role in Fruit Development and Stress Tolerance

**Figure 1:**
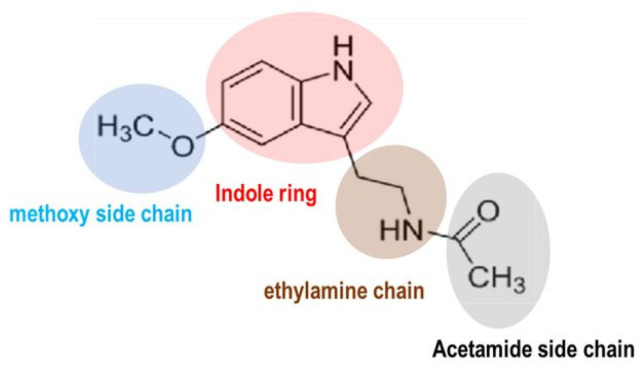
Chemical structure of melatonin (MEL).

Melatonin (MEL) is derived from the amino acid tryptophan. Chemically, MEL is known as *N*-acetyl-5-methoxytryptamine (Figure 1) and is often referred to as the skin-lightening molecule or the ‘darkness hormone’ due to its activation in response to darkness [1].

The occurrence of MEL was first reported in animals and later in plants [2]. However, recent studies have shifted the research focus toward the functional applications of MEL in horticulture, particularly as a natural, low-toxicity tool for improving fruit quality and shelf-life rather than its mere presence in plant tissues. Compared to animals, the scientific literature on plants is very limited. In the Scopus database (accessed in January 2026), 7.6% of MEL-related publications correspond to agricultural sciences, highlighting the emerging but still limited exploitation of MEL in crop systems (https://www.scopus.com).

**Figure 2:**
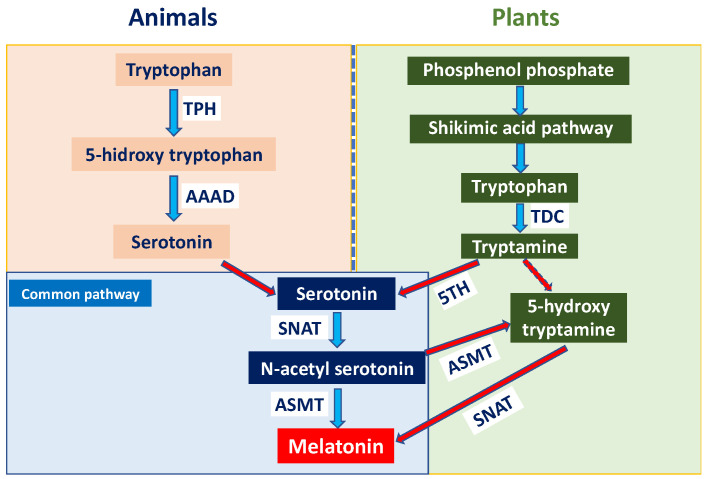
Melatonin (MEL) synthesis in animal and plant cells.

Although melatonin biosynthesis follows a conserved tryptophan-derived pathway in both animals and plants, important differences exist between kingdoms (Figure 2). In animals, melatonin is primarily synthesised in the pineal gland from tryptophan via serotonin and regulates circadian rhythms, sleep, and other physiological functions [3,4]. In plants, tryptophan is synthesised via the shikimate pathway and subsequently converted to tryptamine by tryptophan decarboxylase (TDC). Through the classical route, tryptamine is hydroxylated by tryptamine 5-hydroxylase (T5H) to serotonin, which is acetylated by serotonin *N*-acetyltransferase (SNAT) in chloroplasts and mitochondria and finally methylated by *N*-acetylserotonin *O*-methyltransferase (ASMT) to form melatonin [5]. Alternatively, melatonin can be synthesised via an alternative pathway in which ASMT converts tryptamine to 5-hydroxytryptamine, which is subsequently acetylated by SNAT to produce melatonin in chloroplasts [6]. Recent reports have demonstrated that MEL treatments transcriptionally activate SNAT and ASMT genes in fruit tissues, promoting endogenous MEL accumulation that directly affects fruit senescence and stress tolerance [7,8,9,10,11].

In addition to endogenous biosynthesis, melatonin can be industrially produced using genetically modified microorganisms, including yeast (*Saccharomyces*), bacteria (*Escherichia coli* and *Streptomyces*), lactic acid bacteria (*Lactobacillus* spp.), and algae (*Chlorella*), for pharmaceutical applications [12,13,14].

The occurrence of endogenous MEL has currently been observed in more than 140 plant species, which is known as ‘phytomelatonin’ and is considered a new plant hormone that plays an essential role in many physiological processes, including seed germination, photosynthesis, fruit ripening, postharvest processes, and anti-senescence, among others [15].

**Figure 3:**
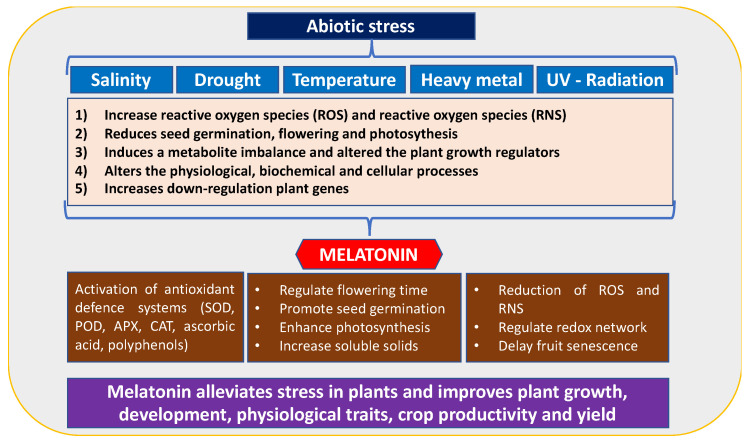
The role of melatonin (MEL) in alleviating various abiotic stresses during plant development.

Abiotic stress is one of the main challenges in agriculture because it notably affects plant growth and yield [16]. Several abiotic stresses, such as temperature fluctuations, salinity, drought, and nutrient deficiency due to climate change, affect agricultural productivity [17]. These stresses have a significant impact on plant development, including seed germination, flowering, and leaf photosynthesis. This leads to reduced plant growth, fruit yield, and quality (Figure 3). Therefore, the effects of these stressors on plant physiology, morphology, and metabiological responses, as well as the determination of defence systems to mitigate the detrimental consequences of abiotic stress, need to be addressed [17].

Over the last decade, the crucial role of MEL in abiotic stress events has been reported, particularly in relation to heat and low temperatures, salinity, drought, and heavy metals, as well as biotic stress caused by bacterial, fungal, and viral contamination. Phytomelatonin regulates the plant redox system by restoring redox homeostasis inducing enhanced abiotic stress resistance [18,19]. MEL can scavenge both reactive oxygen species (ROS) and reactive nitrogen species (RNS) by activating the ascorbate/glutathione cycle and antioxidant enzymes (Figure 3).

The main mechanism by which MEL promotes abiotic stress tolerance is the activation of genes responsible for stress. Mechanistic studies demonstrate that MEL activates mitogen-activated protein kinase (MAPK) signalling cascades which modulate signalling pathways to counteract ROS production by increasing antioxidant enzymes such as peroxidase (POD), catalase (CAT), ascorbate peroxidase (APX), and superoxide dismutase (SOD) [20,21,22,23,24]. Exogeneous preharvest MEL application then induces many physiological and biochemical processes, such as increased chlorophyll and protein content, through the upregulation of associated genes like *CHLG*, *POR*, *CAO*, and proline biosynthesis-related gene, among others, and photosynthesis efficiency via Photosystem-II efficiency and better electron transport rates [25,26,27,28]. Additionally, it is widely reported that MEL activates the phenylpropanoid pathway by upregulating phenylalanine ammonia-lyase (PAL) and chalcone synthase (CHS) pathways, thereby stimulating the biosynthesis of phenolic compounds with strong antioxidant properties that contribute to membrane protection, ROS scavenging, and enhanced stress tolerance [6,15]. Under salinity stress, crop growth and development are limited, which threatens global fruit production. This is mainly due to imbalances in osmolyte and nutritional levels and ROS generation, which affect plant growth [29].

Accordingly, MEL treatment was effective in scavenging photochemical and non-photochemical compounds, thereby increasing net photosynthesis by protecting the photosynthetic apparatus [30]. Another important abiotic stress is drought, which limits crop yield due to the reduced water availability and altered plant physiology [25]. MEL confers tolerance to drought stress by regulating photosynthetic efficiency and quenching ROS via the antioxidant defence machinery [31,32,33].

Cold stress has also been demonstrated to have a devastating impact on plants, thereby limiting crop growth and fruit production, particularly through altering membrane permeability and antioxidant potential [25]. Preharvest MEL treatment enhances ROS scavenging and alleviates cold-induced growth inhibition by improving stomatal conductance and net photosynthesis. It has been related to reductions in malondialdehyde (MDA) and an increase in CAT, POD, and SOD enzyme activities [34]. Heat stress greatly affects plant growth and is considered one of the most important factors affecting food security, resulting in a devastating reduction in fruit yield. MEL supplementation enhances the activity of SOD and APX enzymes, reducing ROS production and attenuating leaf senescence [35]. Ultraviolet (UV) radiation compromises crop production due to its continuous increase in intensity caused by ozone layer depletion. However, the application of MEL is considered potent in alleviating the devastating effects of UV-induced DNA damage by enhancing ROS and RNS production, as has been observed in wild-type apple (*Malus hupehensis*) [36,37]. Overall, MEL is an effective tool for improving plant tolerance to abiotic stresses. It modifies several molecular, physiological, and biochemical processes induced by ROS reduction and the activation of the antioxidant machinery.

Despite significant progress, most available studies remain restricted to single fruit species, specific cultivars, and isolated stress factors. The differential responsiveness of fruit cultivars to MEL, the stability of MEL-induced transcriptional reprogramming during storage, and the effectiveness of MEL under combined abiotic stresses are still poorly understood, representing major barriers for the large-scale adoption of MEL in commercial horticultural systems. Unlike previous general reviews centred mainly on plant stress physiology, the present work integrates mechanistic, physiological, and postharvest perspectives to position MEL as a functional regulator of fruit quality and shelf-life. Furthermore, this review critically synthesises the current knowledge on the role of preharvest MEL in mitigating abiotic stress and on postharvest MEL applications in delaying senescence, preserving organoleptic and nutritive attributes, and enhancing bioactive compounds and antioxidant capacity, thereby highlighting the commercial and economic potential of MEL for sustainable horticulture.

## 2. The Role of Melatonin in Improving Fruit Quality

Fruit ripening is a developmentally regulated process that determines colour, texture, flavour, and aroma and directly influences postharvest life, particularly in climacteric fruits in which ethylene controls the progression of ripening [38,39,40,41,42,43]. Recent evidence indicates that melatonin acts as a key modulator of this process by interacting with ethylene and abscisic acid signalling pathways, thereby regulating the timing and uniformity of ripening and delaying senescence in fruit tissues [6,15,44]. Preharvest melatonin application has been shown to postpone climacteric ethylene peaks, maintain firmness, and preserve chlorophyll and carotenoid balance in several fruits, while postharvest treatments reduce softening and quality loss during storage [6,45].

Melatonin modulates antioxidant metabolism, cell wall-modifying enzymes, and pigment biosynthesis pathways. Through the regulation of ROS homeostasis and transcriptional control of genes associated with ethylene biosynthesis, cell wall disassembly, and phenylpropanoid metabolism, melatonin contributes to improved firmness, colour development, and flavour retention, ultimately enhancing fruit quality and extending shelf-life in both climacteric and non-climacteric fruits [6,15].

### 2.1. Fruit Growth and Development

Global population growth is increasing the demand for high-quality fruit production, highlighting the need for sustainable strategies to preserve fruit quality and nutritional value [46,47]. Melatonin has emerged as a promising natural regulator to improve fruit quality and postharvest performance while reducing reliance on synthetic preservatives [48]. It has been observed that plants subjected to abiotic stress enhance MEL concentration, mainly due to their adaptation to these uneven conditions by triggering plant defence systems. Additionally, MEL content can influence the regulation of fruit growth and development [21]. In non-stressed plants, the endogenous concentration of MEL at harvest depends on the plant species and the cultivar studied (Table 1). MEL modulates plant growth and developmental programmes by regulating key biosynthetic and signalling genes, including the phytomelatonin biosynthesis gene *SNAT2*, and interacting with gibberellin-related pathways through the transcriptional regulation of *ent-kaurene synthase* (KS) and *FLOWERING LOCUS T* (FT) [24,49]. These regulatory modules influence flowering time, cell division, and mesocarp expansion, thereby indirectly determining fruit set, growth rate, and final fruit size. Moreover, MEL has been shown to regulate carbohydrate metabolism and redox homeostasis through its antioxidant metabolites (AFMK and AMK), contributing to the improved carbon–nitrogen balance and photosynthetic efficiency, which are critical determinants of fruit growth and yield [50,51].

Then, the positive role of MEL in fruit ripening has been researched in some reports. For example, the endogenous content of MEL was very low during the early first stage of cherry development, increased during the second stage of development, and decreased further during ripening. This suggests that the endogenous concentration of MEL is greatly dependent on the stage of fruit development [54]. MEL regulates the expression of ripening genes, as ethylene biosynthesis genes (like *ACS* and *ACO*) and transcription factors (*MdREM10*, *MdERF3*) that control ethylene production impact the fruit quality at harvest [15,55].

In ‘Jinyan’ kiwifruit, the application of MEL (at 50, 100, 150, and 200 μM) enhanced the fruit weight in a dose-dependent manner [56]. Similarly, in five apple varieties, both yellow (‘Reineta’ and ‘Golden’) and red apples (‘Teórica’, ‘Sanroqueña’, and ‘Caguleira’) treated with MEL at 500 μM increased fruit mass [57]. Foliar spray of ‘Hongdeng’ sweet cherry with MEL at 50 and 100 μM also improved the fruit weight [42]. These authors revealed that transcript levels of *PacTDC* and *PacSNAT* were greatly correlated to the endogenous MEL concentration, suggesting the pivotal importance of melatonin accumulation in stimulating fruit growth. In ‘Merlot’ grapevines, double treatment with MEL at 100 mg g^−1^ during the pre-veraison stage enhanced the weight of grape berries about 6.6% more [58]. In ‘Zaosu’ pear, preharvest MEL at 100 μM increased the fruit size by 47.85% compared to the control, which was attributed to have enhanced the net photosynthesis rate and maximised the quantum efficiency of photosystem II at the latter stage of fruit development; in addition, this treatment enhanced the sucrose and sorbitol concentrations, due to the improved starch degradation, and the lower expression levels of the invertase gene *Pbinvertase 1/2* and the higher levels of expression of *PbSPS1/2/3* resulted in the activation of the sucrose phosphate synthase activity [59].

### 2.2. Crop Yield and Fruit Quality

The application of preharvest MEL can modulate fruit ripening and enhance yield and quality [60]. For example, immersing tomato seeds in MEL enhances the concentrations of Ca^2+^, lycopene, and vitamin C, while MEL irrigation increases the levels of total soluble solids (TSS), organic acids, and lycopene in tomato plants. Similarly, MEL treatment of pear trees increased fruit weight, probably due to an increased net photosynthetic rate during the final fruit development stage, as well as an increased TSS content, especially of sucrose and sorbitol. Preharvest foliar spraying of grapes at the veraison stage enhances berry coloration by stimulating genes related to anthocyanin synthesis and increasing the endogenous MEL concentration, while reducing unripe and overripe bunches [61]. In sweet cherries, endogenous MEL content showed a negative correlation with fruit maturation; however, preharvest MEL treatment modulated fruit ripening and enhanced quality attributes at harvest [10,62]. Interestingly, preharvest treatment with MEL significantly improved the weight of sweet cherries, their TSS content, and their total phenolic compounds, including total anthocyanins, as well as their antioxidant activity [10,63].

Several reports have reviewed the effect of MEL on fruit yield and quality [15,21,45,64]. Accordingly, preharvest treatment of lemon fruit with MEL at concentrations of 0.1, 0.3, and 0.5 mM enhanced yield in terms of kg per tree and number of fruits per tree. Yield increased by 25–30%, particularly at a concentration of 0.1 mM, while fruit weight was highest at a concentration of 0.5 mM [65]. Additionally, the number of culled fruits was reduced for all MEL-treated lemons. Moreover, MEL treatments increased endogenous melatonin and flavanone content, enhancing fruit quality and health-promoting benefits [11]. In sweet cherries, all concentrations of MEL enhanced the crop yield. This effect was due to a higher number of fruits harvested per tree. Yield increased by between 45% and 55%, especially with MEL at 0.3 mM. Fruit weight increased with all MEL treatments, especially with MEL at 0.5 mM [66]. In ‘Primetime’ Japanese plum, during MEL treatment at 0.1, 0.3, and 0.5 mM for two consecutive years, the three preharvest applications versus two, independently of the doses applied, increased the endogenous MEL content and in turn improved fruit quality, such as higher fruit firmness, colour, TSS, and TA [67]. In tomato, MEL (at 0, 50, and 100 μM) showed that the best concentration was 100 μM by promoting the sugar accumulation due to enhancing the sucrose synthase and sucrose phosphate synthase acid convertase enzyme activities. Other parameters, such as the ten amino acids, six phenolic acids, three flavonoids, and volatile compounds, were also enhanced by MEL at 100 μM [68]. In chilli pepper, MEL at 50 and 100 μM increased the total yield by 17.44% mainly due to the enhanced fruit weight and fruit weight per plant. Related to fruit mass, a 9.33 and 5.94 g increase was recoded for MEL at 100 and 50 μM, respectively [69].

Pomegranate trees treated with 0.1 mM MEL increased the yield per tree, the number of fruits per tree, and the fruit weight. This is the first report of the use of MEL as a preharvest treatment to increase crop yield [70]. Another positive effect of preharvest MEL treatment was its impact on fruit cracking in two sweet cherry cultivars (‘Prime Giant’ and ‘Sweetheart’). Foliar applications of MEL at 0.01, 0.05, and 0.1 mM reduced cracking in both cultivars, although the effect depended on the cultivar and ripening stage [71]. In pomegranate trees, 0.1 mM doses of MEL were applied as a preharvest foliar spray. The results showed that concentrations of both organic acids and reducing sugars, as well as aril colour, were enhanced in MEL-treated fruits. Furthermore, MEL at 0.1, 0.3, and 0.5 mM increased quality parameters such as firmness, TSS, and TA. Additionally, the content of bioactive compounds (total phenolics and total anthocyanins) and antioxidant activity were enhanced by MEL applications.

**Figure 4:**
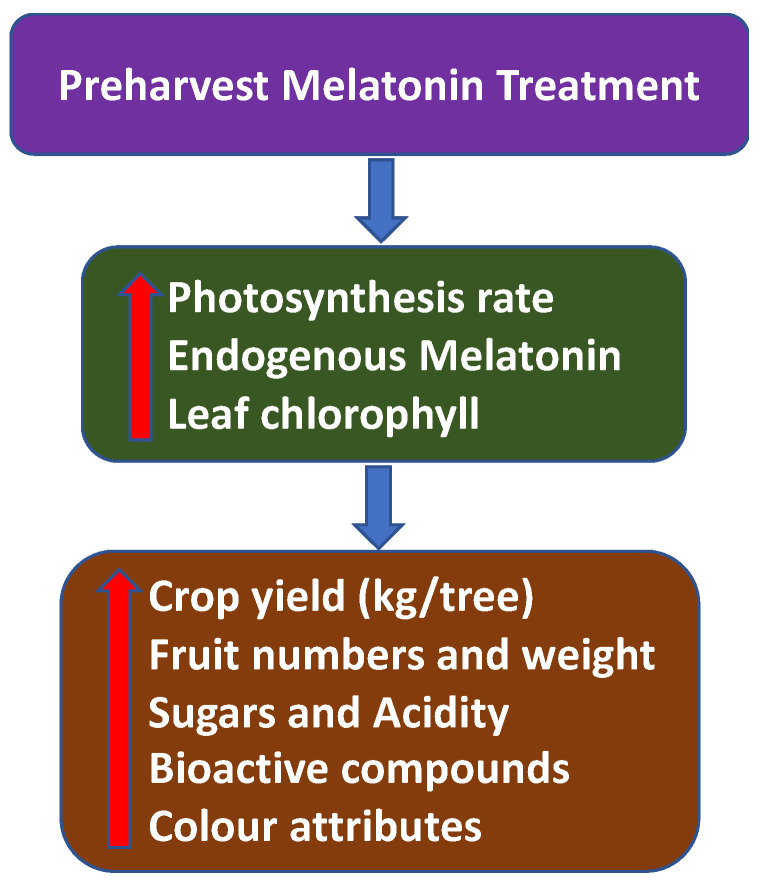
The effect of preharvest melatonin (MEL) treatment on crop yield and quality attributes of several fruit and vegetables at harvest.

Overall, the preharvest application of MEL will increase photosynthesis and the chlorophyll concentration in the plant, as well as enhancing endogenous MEL. This will improve crop yield and quality at harvest (Figure 4).

## 3. Melatonin and Postharvest Quality of Fruits

The unique flavour and nutritional components of fruit, such as sugars, organic acids, phenolic compounds, and vitamins, make it a popular dietary choice worldwide. However, fruits are highly perishable by nature, which limits their storability due to quality loss. The scientific community has always been concerned about fruit preservation tools during postharvest storage and has been looking for new, environmentally friendly, sustainable tools [72]. Among these new tools, MEL has emerged as a potent molecule for maintaining postharvest fruit quality, extending shelf-life, and alleviating chilling injury (CI) [73]. Accordingly, preharvest treatment with 0.5 mM MEL increased the endogenous MEL concentration in sweet cherries by 3.6-fold compared with control fruits at harvest. Furthermore, the combination of pre- and postharvest MEL treatment and cold storage induced the highest endogenous MEL content, which was 7.5 times higher than the control group [10]. This increase could be related to higher MEL absorption due to postharvest and preharvest application, although the activation of the MEL biosynthetic pathway could not be confirmed. Several reports have demonstrated that MEL treatments would upregulate the genes responsible for MEL biosynthesis, such as SNAT, *TDH*, *TDC*, *5TH*, and *ASMT* enzyme activities, thus increasing endogenous MEL [64,74,75].

During postharvest storage, the limiting factors are the incidence of CI and decay caused by fungal and bacterial growth. Disruption to the integrity of the cell membrane and enhanced electrolyte leakage (EL) are clear symptoms of fruit deterioration and senescence. These effects are accompanied by exacerbated ROS and RNS generation and induced lipid peroxidation, which stimulates fruit senescence and deterioration [38]. In recent years, a large body of research has investigated the role of MEL in regulating postharvest ripening and senescence, particularly in climacteric fruits, improving fruit quality and storability [64,76]. In red bananas, postharvest treatment with 1 mM MEL inhibited ethylene production and respiration rates and reduced softening by inhibiting cell wall-degrading enzymes such as polygalacturonase (PG), pectin methyl esterase (PME), and β-glucosidase. Additionally, antioxidant enzymes (CAT, SOD, and POD) were enhanced by MEL treatment [77]. Similarly, MEL at 10 μM significantly reduced strawberry ripening by delaying anthocyanin content and lowering TSS while increasing TA and phenolic compounds, as well as enhancing PAL and antioxidant activity. The reduction in strawberry ripening was attributed to enhancing ABA biosynthesis [78].

In general, MEL plays a crucial role in enhancing the proteins related to the inhibition of apoptosis and the biosynthesis of anthocyanins during fruit ripening. Fruit quality is also enhanced by increasing TSS and reducing firmness losses. MEL is also known to increase in bioactive compounds (polyphenols and anthocyanins) and the antioxidant capacity, as well as enhancing the antioxidant enzymes responsible for scavenging the ROS and RNS [73]. Table 2 shows the optimal MEL concentrations and storage conditions for various fruit commodities, along with their primary effects on fruit quality, ripening, senescence, and shelf-life.

Postharvest MEL treatment of guava fruit reduced softening and the respiration rate, as well as weight loss, while maintaining TSS and TA [89]. MEL application enhanced colour attributes by increasing total anthocyanins and individual anthocyanins. In nectarines, MEL concentrations of 250, 500, and 1000 μM maintained the visual appearance and health-promoting compounds while extending shelf-life [90]. Preharvest application of 1 mM MEL resulted in enhanced flavanone content in the juice at harvest; this increase was higher after postharvest MEL application. Combined pre- and postharvest treatments resulted in a further 27% increase in flavanone content. These results are attributed to higher endogenous MEL levels, making it a promising strategy for enhancing the functional quality of lemons [11]. Moreover, melatonin treatments at the pre- or postharvest stage, or in combination, promoted the accumulation of endogenous melatonin, flavanones, anthocyanins, and vitamin C in blood oranges. Thus, melatonin elicitation emerged as an effective strategy for improving the functional quality of blood oranges [9].

Low-temperature storage is widely used to extend shelf-life. However, tropical and subtropical fruits are highly susceptible to CI development. CI starts with the modification of the plasma membrane, transforming the liquid crystalline phase into the gel phase and increasing the EL and malondialdehyde (MDA). CI symptoms include abnormal ripening, surface pitting, hardening of the flesh, and browning of the peel and flesh, although these depend on the species and cultivars of fruit [91]. The efficacy of MEL application in reducing CI has been reported for a wide range of fruit species, although it seems to vary depending on cultivar, genotype, and storage conditions.

**Figure 5:**
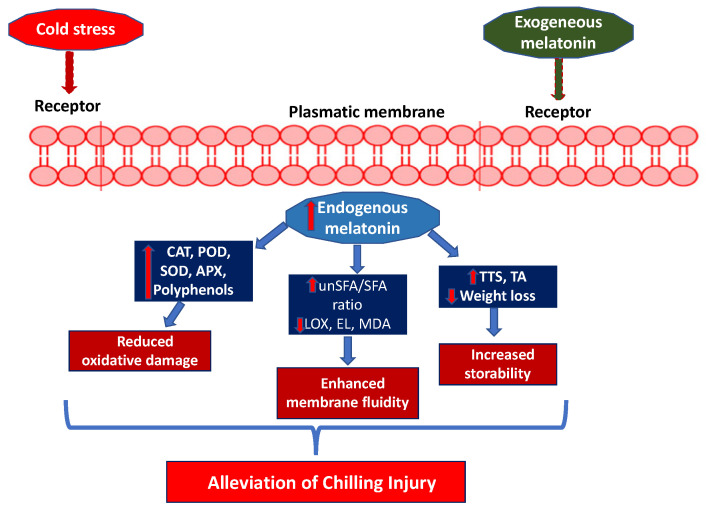
A proposed mechanism for reducing chilling injury (CI) after postharvest treatment with melatonin (MEL).

Exogenous MEL treatment can improve the storability and fruit quality by maintaining firmness, TSS, and TA, delaying physiological weight loss and alleviating CI [92]. During storage of sweet cherries, a reduction in endogenous MEL has been observed. This decrease could be related to MEL’s ability to scavenge ROS generated under chilling stress conditions. Under these conditions, MEL increases the activity of antioxidant enzymes such as SOD, CAT, and APX [44]. The decrease in endogenous MEL could also be attributed to the advancement of the ripening process in sweet cherries, which has been associated with MEL consumption in fruits (Figure 5) [62].

Postharvest treatment with 50, 100, 150, or 200 μmol L^−1^ of MEL alleviated the symptoms of CI in guava fruit by maintaining cell membrane permeability and ascorbic acid levels and preventing browning in both the peel and flesh. Of all the MEL concentrations, 100 μmol L^−1^ produced the best quality characteristics and the lowest CI index [89]. In cherimoya fruit, MEL at concentrations of 0.01, 0.05, and 0.1 mM, when applied as dips, reduced CI symptoms by delaying ethylene production and EL and enhancing colour retention while improving TSS, TA, and the total phenolic content and antioxidant activity in the peel [85]. Similarly, postharvest MEL treatment reduced weight loss and maintained TSS and TA in ‘Colorado’ and ‘Mikado’ cultivars, delaying CI symptoms [93]. Postharvest MEL treatment at 100 μM in several mango cultivars showed alleviation of CI symptoms, which was associated with increased levels of the antioxidant enzymes SOD, CAT, POD, and APX, as well as increased antioxidant capacity [82].

The reduction in weight loss following MEL treatment could be attributed to a decrease in metabolic activity, leading to lower energy consumption and reduced transpiration and respiration rates in stored fruit [90]. Since the respiration rate is due to the consumption of both organic acids and sugars, the lower weight loss resulting from MEL application justifies the higher TSS and TA content [94]. In relation to CI damage, fungal growth occurs, leading to fruit decay. Postharvest MEL treatment has been observed to reduce the incidence of decay caused by *Botrytis cinerea* (grey mould), *Penicillium expansum* (blue mould), and *Colletotrichum gloeosporioides* (anthracnose decay), among others [6]. In citrus fruits, the attenuation of fungal decay by exogenous MEL treatments could be attributed to the accumulation of endogenous MEL, which minimises the increase in the radical O_2_^−^ and the accumulation of H_2_O_2_. At the same time, it enhances the antioxidant enzymes SOD, CAT, and APX, as well as increasing the content of phenolic compounds and total antioxidant activity [95].

## 4. Melatonin and Its Cross-Talk with Other Plant Hormones in Response to Abiotic Stress

**Figure 6:**
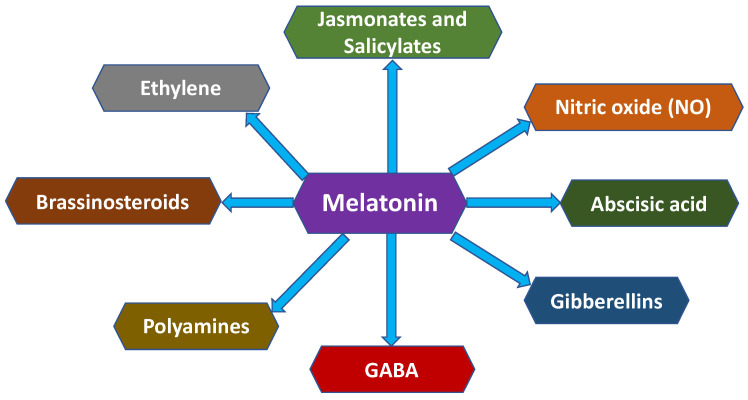
Cross-talk between melatonin (MEL) and other plant hormones.

The ability of melatonin (MEL) to improve fruit quality before and after harvest is largely mediated through its coordinated interaction with other phytohormones involved in stress perception, ripening control, and senescence regulation, such as polyamines (PAs), nitric oxide (NO), abscisic acid (ABA), gamma-aminobutyric acid (GABA), and ethylene, among others (Figure 6) [44]. These hormone networks integrate stress signalling with metabolic and structural processes that determine fruit firmness, colour development, antioxidant capacity, and shelf-life stability.

The role of PAs, namely putrescine (PUT), spermidine (SPD), and spermine (SPM), in plant chilling tolerance and CI-injured fruit tolerance during storage has been reported during plant stress tolerance [96]. Postharvest treatment with MEL induces the stimulation of both ornithine decarboxylase (ODC) and arginine decarboxylase (ADC), which are the main enzymes involved in PA biosynthesis [97,98,99]. In this sense, MEL application enhances endogenous PA levels, as observed in peach and cucumber [92]. Furthermore, postharvest treatment with MEL induced a CI defence mechanism in mango, accompanied by a reduction in EL and ROS [82]. The accumulation of PAs stabilises membrane phospholipids, reduces electrolyte leakage, and delays softening, directly contributing to firmness retention and reduced CI incidence in stored fruits.

MEL induces the NO signal by enhancing the expression of nitric oxide synthase genes. In turn, an increase in endogenous NO is observed, which stimulates the formation of PAs and proline due to the accumulation of H_2_O_2_ as a signal [92]. Both MEL and NO are involved in plant physiology, acting independently on molecular and biochemical pathways, although they can also exhibit cross-linked interactions in certain circumstances. Accordingly, NO can be used as a donor to form *N*-nitrosomelatonin, a recently discovered nitrous form of melatonin with promising effects on plant physiology, especially regarding RNS formation [100].

ABA, also known as the ‘universal stress hormone’, has been reported to play an essential role in many physiological processes in response to several abiotic stresses through increased endogenous ABA levels. Thereby, the relationship between MEL and ABA has gained increasing scientific interest, which depends on the dose of MEL, the plant species, and the type of stress [60]. ABA stimulates the genes responsible for synthesising antioxidant enzymes, improving the scavenging of ROS. Under abiotic stress, especially cold stress, MEL stimulates endogenous ABA content, whereas under salinity and drought stress, MEL suppresses this increase. It is worth noting that both MEL and ABA can regulate stomatal closure differently [101]. MEL can close stomata at low concentrations, while ABA induces stomata opening at a wide range of doses, suggesting different mechanisms of stomatal closure dynamics and thus different regulations of abiotic stress. This hormonal reprogramming contributes to improved firmness retention, delayed peel browning, and enhanced antioxidant status during storage.

Ethylene is the only gaseous plant hormone involved in many physiological processes, including fruit ripening, senescence, and resistance to different stresses [102]. Fruit ripening involves a wide range of coordinated physiological effects that result in changes to the colour, flavour, aroma, and nutritive compounds of climacteric fleshy fruits. Accordingly, exogenous MEL plays a crucial role in the ripening and quality of tomato fruits by enhancing ethylene biosynthesis [103]. Indeed, MEL affects the activity of key enzymes in ethylene production, including ACC synthase (ACS) and ACC oxidase (ACO) [104]. MEL treatment of pak choi reduced postharvest leaf yellowing by decreasing both ethylene and respiration rates, improving visual and nutritional quality during storage [105].

The upregulation of the endogenous concentrations of JA by postharvest MEL has been reported to alleviate CI and softening for several fruits. JA concentrations increased due to abiotic stress and thus contributed to stress tolerance [106]. In this sense, JA is involved in plant defence responses, as well as flowering and fruit formation. However, the relationship between JA and MEL displays both synergistic and antagonistic behaviours depending on species and stress context. For example, in *Arabidopsis thaliana*, MEL suppressed the 20-fold increase in anthocyanins induced by JA by negatively regulating their biosynthetic pathways [107]. Moreover, transgenic lines overexpressing the melatonin biosynthetic gene SNAT exhibited reduced JA-induced anthocyanin accumulation, further supporting an antagonistic MEL–JA interaction at the transcriptional level [102]. Conversely, in watermelon, MEL treatment increased endogenous methyl jasmonate (MeJa) levels, which in turn promoted cold acclimation, suggesting a synergistic MEL–JA interaction under low-temperature stress conditions [108]. These apparently contradictory responses indicate that MEL can either suppress or potentiate JA signalling depending on the developmental stage, hormonal dose, species specificity, and type of abiotic stress.

Plant immunity and defence responses are controlled by salicylic acid (SA) in response to abiotic stress, and MEL application is known to interact with SA by affecting its signalling [108,109]. Furthermore, MEL increases the expression of key genes responsible for SA biosynthesis, such as isochorismate synthase and PAL, since chorismate serves as an SA precursor. Accordingly, MEL treatment increased endogenous SA in kiwifruit, probably due to the upregulation of SA-responsive PAL genes [110]. In another study, exogenous MEL was found to enhance PAL activity, which in turn increased the SA concentration, leading to reduced membrane lipid peroxidation and alleviating CI [96]. This interaction stimulates phenolic accumulation, improves antioxidant capacity, and reduces membrane lipid peroxidation, thereby enhancing the functional quality and storability of fruits through CI alleviation.

γ-Aminobutyric acid (GABA), as melatonin, is a non-protein amino acid which was first described as a cellular messenger. GABA is synthesised from glutamate, catalysed by the enzyme glutamate decarboxylase, but also via PAL metabolism. GABA has been reported to mitigate plant abiotic stress and has been postulated as a potent tool for improving fruit quality during postharvest storage by enhancing the capacity to scavenge ROS [111,112]. Several studies have demonstrated that both MEL and GABA reduce the detrimental effects of abiotic stress independently. However, when plants are under stress, simultaneous treatment with melatonin and GABA results in greater benefits because they act synergistically [113]. Another study showed that MEL mitigates the adverse effects of stress, with GABA playing a key role in alleviating them. Thus, the application of exogenous MEL improved the yield of kiwifruit subjected to flooding stress [114]. This close relationship between both phytohormones is essential to ensure fruit quality preservation.

**Figure 7:**
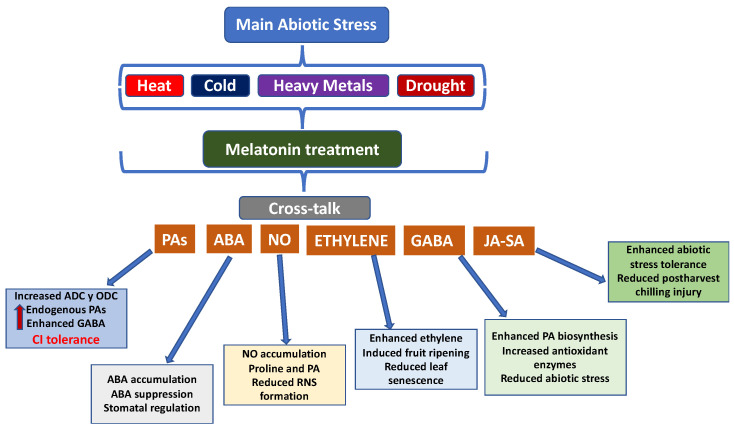
Cross-talk between melatonin (MEL) and other plant hormones, and the related effects.

Overall, these studies demonstrate that MEL plays a central role in modulating multiple plant hormones and reducing the concentration of ROS caused by abiotic conditions, delaying senescence and preserving fruit quality during both pre- and postharvest stages. This has implications for both plant biology and agriculture (Figure 7).

## 5. Conclusions and Future Trends

This review provides an update on the mechanisms by which MEL improves plant tolerance to abiotic stress and enhances fruit quality. Exogenous MEL, when applied either pre- or postharvest, is a promising strategy for enhancing antioxidant enzymes by scavenging ROS production, which affects both crop yield and fruit quality at harvest time. Promoting the antioxidant enzymes SOD, POD, CAT, and APX helps plants cope with abiotic stress. Although MEL has proven to enhance chilling tolerance in low-temperature storage in horticultural products, CI sensitivity and symptoms affect several fruits and vegetables differently.

Unlike the ‘classical’ plant hormones, MEL plays a significant role in anti-stress responses, although effective MEL concentrations vary greatly between plant species and even between cultivars within the same species. The question of the application of MEL at higher concentrations remains unclear since several MEL binding sites occur in the cell membrane and signal transduction exists. Although the existing literature demonstrates the positive role of MEL in regulating fruit growth, development, ripening, and quality at harvest time and during postharvest storage in conjunction with plant hormones, more studies are necessary, especially those related to omics, to clarify the transcription factors involved in regulating the signalling cascade.

In this context, future research should prioritise the application of transcriptomic approaches to identify melatonin-responsive transcription factor families, like NAC and MYB, which are known to play pivotal roles in fruit ripening, senescence, and stress tolerance. In parallel, metabolomic profiling should be employed to unravel the association networks between MEL and secondary metabolites, including phenolic compounds, carotenoids, and flavonoids, thereby linking transcriptional regulation with functional quality traits.

The potential of using MEL in horticulture for managing abiotic stress, which affects crop yield, deserves further investigation. Research should focus on elucidating the mechanism of action of MEL when applied either pre- or postharvest. Moreover, given the relationship between MEL and CI, further research is needed into both the molecular and physiological pathways, especially the role of plasma proteins.

Finally, future perspectives should also explore the combined application of MEL with other postharvest preservation technologies, such as controlled and modified atmosphere storage, edible coatings, and nanomaterial-based delivery systems, to enhance its stability, bioavailability, and synergistic efficacy in maintaining fruit quality and extending shelf-life.

## Figures and Tables

**Table 1:** Some examples of endogenous melatonin (MEL) found in various fruits. Adapted from Huang et al. [52] and Ze et al. [53], along with recent research of Garrido-Auñón et al. [9,10] and Agulló et al. [11].

Fruit Name	Cultivar/Species	Tissue	Amount (ng g^−1^) *
Apple	‘Golden Delicious’	Flesh	0–20
Banana	*Musa ensete*	Flesh	0.65
Blood Orange	‘Sanguinelli’	Juice	0.14 **
Cranberry	*V. macrocarpon*	Fruit	97
Cucumber	*Cucumis sativus*	Fruit	0.59
Date palm	‘Medjoul’	Flesh	0.85–4.26
Kiwifruit	*Actinidia deliciosa*	Flesh	0.02
Lemon	‘Fino’	Juice	0.21 **
Mango	*Mangifera indica* Linn.	Juice	0.7
Orange	‘Navel late’	Juice	4.5–20
Papaya	*Carica papaya* L.	Juice	0.24
Pomegranate	*Punica granatum*	Fruit	0.17
Grape	‘Moldova’	Fruit	1.93–7.02
Sweet cherry	‘Prime Giant’	Fruit	4.36–13.95
	‘Sunburst’	Fruit	0.02 ***
Strawberry	‘Montmorency’	Fruit	5.57–19.60
Tomato	*Lycopersicon esculentum*	Fruit	0.032–14.23
Pineapple	*Ananas comosus*	Fruit	0.036

* ng g^−1^ in dried weight (DW) basis. ** ng mL^−1^ in fresh weight (FW) basis. *** ng g^−1^ in fresh weight (FW) basis.

**Table 2:** The effect of melatonin (MEL) at different concentrations and storage conditions on fruit quality and antioxidant activity.

Fruit	Best Concentration	Storage Conditions	Effects	References
Sweet cherry	100 μM	0 °C for 45 days	Delayed senescence and improved the antioxidant potential.	[79]
Sweet cherry	0.3 mM	2 °C for 28 days	Enhanced antioxidant enzymes and delayed the ripening process.	[66]
Sweet cherry	0.5 mM	2 °C for 21 days	Increased endogenous melatonin, anthocyanins, and hydroxycinnamic acids.	[10]
Blueberry	1000 μM	5 °C for 3 weeks	Increased the production of secondary metabolites and antioxidant activity.	[80]
Mango	1000 μM	15 °C for 4 weeks	Increased antioxidant enzymes (CAT and POD).	[81]
Mango	100 μM	5 °C for 28 days	Increased antioxidant activity and enzymes.	[82]
Peach	0.1 mM	4 °C for 28 days	Improved antioxidant activity and GABA biosynthesis.	[83]
Gooseberry	300 μM	10 °C for 21 days	Increased the total phenolic, carotenoid, and antioxidant capacity.	[84]
Cherimoya	0.05 mM	7 °C + 2 days at 20 °C	Delayed ethylene production, increased total phenolics.	[85]
Pomegranate	0.1 mM	10 °C for 90 days	Reduced weight loss and softening.	[86,87]
Lemon	1 mM	2 °C for 21 days	Reduced weight loss and softening.	[88]
Lemon	1 mM	1 °C for 21 days	Increased flavanones and endogenous melatonin.	[11]
Blood orange	1 mM	1 °C for 21 days	Increased anthocyanins, flavanones, vitamin C, and endogenous melatonin.	[9]

## Data Availability

No new data were created or analyzed in this study.
